# A conceptual learning analysis of paired after action and intra action reviews for health emergencies

**DOI:** 10.1002/lrh2.10447

**Published:** 2024-08-29

**Authors:** Elliot Brennan, Seye Abimbola

**Affiliations:** ^1^ Sydney School of Public Health University of Sydney Sydney New South Wales Australia

**Keywords:** AAR, action reviews, COVID‐19, emergency, health system, IAR, learning

## Abstract

**Background:**

Processes of self‐reflection and the learning they allow are crucial before, during, and after acute emergencies, including infectious disease outbreaks. Tools—such as Action Reviews—offer World Health Organization (WHO) member states a platform to enhance learning. We sought to better understand the value of these tools and how they may be further refined and better used.

**Methods:**

We searched the publicly available WHO Strategic Partnership for Health Security website for paired reports of Action Reviews, that is, reports with a comparable follow‐up report. We complemented the paired action reviews, with a literature search, including the gray literature. The paired action reviews were analyzed using the “Learning Health Systems” framework.

**Results:**

We identified three paired action reviews: Lassa Fever After Action Reviews (AARs) in Nigeria (2017 and 2018), COVID‐19 Intra‐Action Reviews (IARs) in Botswana (2020 and 2021), and COVID‐19 IARs in South Sudan (2020 and 2021). Action Reviews allowed for surfacing relevant knowledge and, by engaging the right (in different contexts) actors, asking “are we doing things right?” (single loop learning) was evident in all the reports. Single loop learning is often embedded within examples of double loop learning (“are we doing the right things?”), providing a more transformative basis for policy change. Triple loop learning (“are we learning right”?) was evident in AARs, and less in IARs. The range of participants involved, the level of concentrated focus on specific issues, the duration available for follow through, and the pressures on the health system to respond influenced the type (i.e., loop) and the effectiveness of learning.

**Conclusion:**

Action Reviews, by design, surface knowledge. With favorable contextual conditions, this knowledge can then be applied and lead to corrective and innovative actions to improve health system performance, and in exceptional cases, continuous learning.

## 1 INTRODUCTION

Good governance requires self‐reflection to adapt and remain agile in response to ongoing societal, environmental, political, and economic changes.^1^, ^2^ In health systems, such processes of self‐reflection and the learning they allow are perhaps no more crucial than during acute emergencies that require containment of infectious disease, and especially in fragile, conflict‐affected, and vulnerable countries. These processes may be used to test the health of governance structures and core capacities, including how national and subnational units of government work together to optimize efficiency, equity, and resilience.^3^

There are various processes for health system self‐reflection which at their most effective aim for health system learning at the national and subnational levels, and at institutional, team, or individual levels. A body of emerging work attempts to support improved learning in health systems^4^, ^5^, ^6^ and set out frameworks and strategies to inform better learning practices of health systems and their actors. Learning improves health systems' functions, adaptation and innovation, and resilience. Learning may occur through three means—Information, Deliberation, and Action.^4^ WHO's mandate supports collaboration in health system learning across these different means of learning.

Course development, disease‐specific information sessions, workshops, simulation exercises, Early Action Reviews (EARs), Intra‐Action Reviews (IARs), and After‐Action Reviews (AARs) all support these means of learning in health systems.^7^, ^8^, ^9^, ^10^ Learning requires and promotes broader connections within a health system and collective understanding among its actors. Existing tools under the International Health Regulations Monitoring and Evaluation Framework (IHR MEF)^11^ offer various voluntary tools for member states to enhance their learning and evaluate their overall readiness for a health emergency. The establishment of new IHR MEF, after the acute phase of the COVID‐19 pandemic, and emerging tools to support better health emergency preparedness, response, and resilience provide an opportunity to further refine these tools.

These tools for self‐reflection to allow health system learning include Action Reviews; the first form of which were AARs. An AAR is a qualitative review at the end of an emergency of what occurred during the emergency response to identify best practices, gaps, and lessons learned. The modern AAR emerged through use by the US Army during World War II and has since been used systematically as a means to discuss an event, what happened, why, and uncover specific lessons learned.^12^ WHO has adopted AARs as a platform for learning from public health events and outbreaks. A 2019 review of AARs found that an AAR “*contributes to a culture of continuous personal, collective and institutional learning aimed at the gathering of lessons learned and best practices*.”^13^, ^14^ These lessons learned and best practices may then be stored in a “lessons learned database” to provide long‐term institutional memory.^13^

**FIGURE 1:**
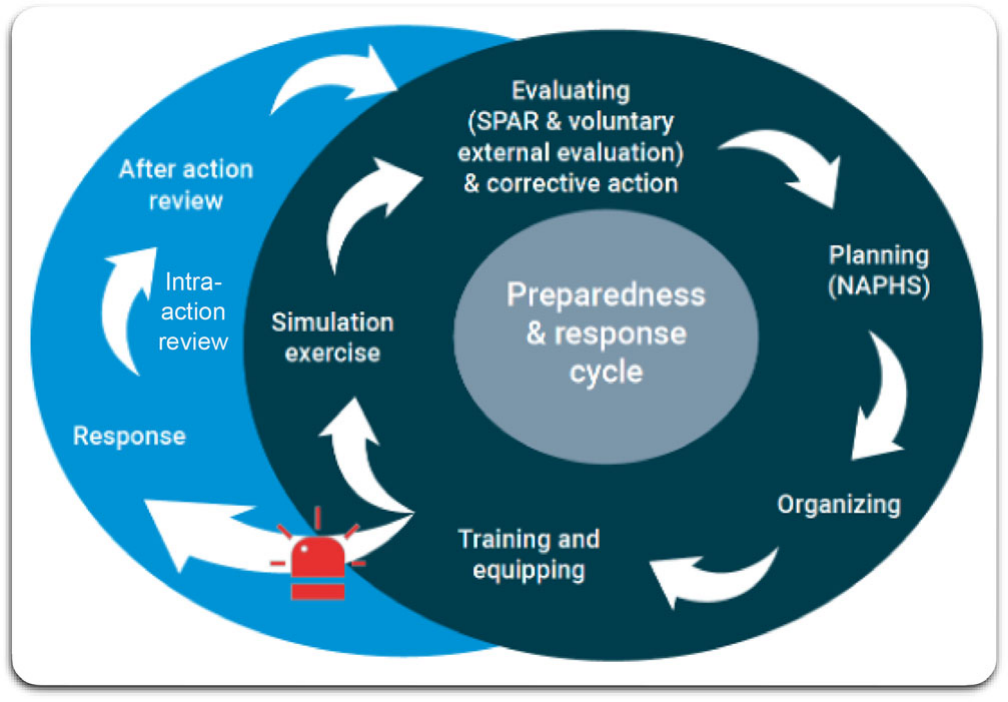
The relationship between action reviews and the preparedness and response cycle. Adapted from preparedness and response cycle.^8^

The WHO Guidance on AARs^13^ recommends four potential formats for AARs (see Figure 1). The formats are an after action debrief, key informant interviews, working group format, and a format that combines any or all of the three.^13^ The choice of a format may depend on the aspects of a response being evaluated, the availability of participants, time or funds available, and the overall complexity of the event. The flexibility of formats provides agility for countries to implement an AAR within 3 months after the end of an outbreak or public health event.

Building off AARs, another form of Action Review, IARs emerged from the need during the COVID‐19 pandemic to learn lessons during a protracted health emergency.^7^ IARs are a qualitative review during an emergency response to identify best practices, gaps, and lessons learned to provide corrective actions going forward. The IARs were developed such that lessons gleaned through IARs aimed to provide insight for countries to course correct and refine their response to the ongoing health emergency. In 2023, WHO guidance for EARs was published^15^ to address the need for rapid assessment and intervention at the outset of a public health event.

Unlike Simulation Exercises—another voluntary tool under the IHR MEF—Action Reviews allow health systems to assess how preparedness systems work under a real health emergency.^13^, ^14^ Action Reviews may be incorporated into national strategic plans to improve health emergency preparedness, response, and resilience. They can be recorded through mandatory reporting, under IHR MEF, of the State Parties self‐assessment annual reporting (SPAR) and may be included in voluntary Joint External Evaluation (JEE) and National Action Plans for Health Security (NAPHS).^16^ Action Reviews may find particular usefulness and impact when the event itself may be a reoccurring or seasonal disease outbreak or a protracted health emergency, such as Lassa Fever or COVID‐19.^12^

A conceptual analysis aims to reduce concepts into their parts to improve understanding of individual components. This conceptual analysis explores learning in health systems after or during health emergencies. It does this using information within existing paired comparable reports of AARs and, separately, paired comparable reports of IARs. We analyze, using the “Learning Health Systems” framework developed by Sheikh and Abimbola,^4^ how, as a platform for self‐reflection, health system learning occurs through the reviews, and also, when, how, why, and where such learning occurs. The aim of this analysis was to better understand the value of these tools and how they may be further refined and better used.

## 2 METHODS

### 2.1 Search strategy

#### 2.1.1 Action review search

We searched WHO's Strategic Partnership for Health Security (SPH) website to identify publicly available AAR and IAR reports. We sought to identify two comparable reports of the review of the same public health emergency conducted in the same country. Three case studies of paired Action Reviews were identified (see Table 1): Lassa Fever AARs in Nigeria (2017 and 2018), COVID‐19 IARs in Botswana (2020 and 2021), and COVID‐19 IARs in South Sudan (2020 and 2021). Below are the AAR and IAR reports available for this analysis with the identified response pillars.

**TABLE 1:** Paired action reviews.

Title	Link	Source	Country‐selected Pillars
Ministry of Health, South Sudan, Country COVID‐19 Intra‐Action Review Report, November 2020.	https://cdn.who.int/media/docs/default‐source/health‐security‐preparedness/cer/iar/south‐sudan‐‐‐covid‐19‐intra‐action‐review.pdf?sfvrsn=de995e23_3&download=true	Government	Pillar 1: Country‐level coordination, planning and monitoring
			Pillar 2: Risk communication and community engagement
			Pillar 3: Surveillance, rapid‐response teams, and case investigation
			Pillar 4: Points of entry
			Pillar 5: National laboratories
			Pillar 6: Infection prevention and control
			Pillar 7: Case management
			Pillar 8: Operational support and logistics
Ministry of Health, South Sudan, Country COVID‐19 Intra‐Action Review Report, August 2021.	https://extranet.who.int/sph/sites/default/files/document‐library/document/IAR%20COVID‐19%20South%20Sudan%20%2805‐06%20Aug%202021%29.pdf	Government	Pillar 3: Surveillance, rapid‐response teams, and case investigation
			Pillar 5: National laboratories
			Vaccination
Ministry of Health and Wellness, Botswana, Country COVID‐19 Intra‐Action Review Report, November, 2020	https://extranet.who.int/sph/sites/default/files/document‐library/document/IAR%20COVID‐19%20Botswana%20%2809‐12%20Nov%202020%29.pdf	Government	Pillar 1: Country‐level coordination, planning and monitoring
			Pillar 2: Risk communication and community engagement
			Pillar 3: Surveillance, rapid‐response teams, and case investigation
			Pillar 4: Points of entry
			Pillar 5: National laboratories
			Pillar 6: Infection prevention and control
			Pillar 7: Case management
			Pillar 8: Operational support and logistics
			Pillar 9: Maintaining essential health services and systems
Ministry of Health and Wellness, Botswana, Country COVID‐19 Intra‐Action Review COVID 19 Vaccination Report, April, 2021	https://extranet.who.int/sph/iar‐details/31493	Government	COVID‐19 vaccination as per the National Deployment and Vaccination Plan (NDVP)
Nigeria Centre for Disease Control, AAR Lassa Fever Nigeria, June 2018	https://extranet.who.int/sph/sites/default/files/document‐library/document/AAR%20Lassa%20Fever%20Nigeria%20%285‐7%20June%202018%29.pdf	Government	Coordination and Logistics Case management, Safe Burial and IPC Surveillance Risk Communication and Social Mobilization Laboratory
Nigeria Centre for Disease Control, AAR Lassa Fever Nigeria, August 2017 (August 2, 2017)	https://extranet.who.int/sph/sites/default/files/document‐library/document/AAR%20Lassa%20Fever%20Nigeria%20%2821‐22%20Aug%202017%29.pdf	Government	Coordination Case Management and IPC Surveillance Laboratory Logistics Risk Communication

### 2.2 Literature search

A keyword search was conducted in August 2023 by EB, in consultation with SA, on three databases: Ovid Medline, SCOPUS, and Global Health. The selection of the databases to search was informed by discussions with colleagues working in the relevant fields. First, a multi‐field search of the Global Health database was conducted using the keywords *Lassa AND Fever AND Nigeria AND (after action OR after‐action)*. The time frame search was through to August 2023. This returned 1 result.^17^ Second, a multi‐field search of Ovid Medline (R) was conducted using *Lassa AND Fever AND Nigeria AND (after action OR after‐action)*. The time frame search was through to August 2023. This returned 1 result.^17^ Third, a multi‐field search of SCOPUS was conducted using the keywords *Lassa AND Fever AND Nigeria AND (after action OR after‐action)*. The time frame search was through to August 2023. This returned 427 results. After a review of titles and abstracts, and where necessary texts, four articles were selected.^16^, ^17^, ^18^

This process was then repeated for keywords *COVID‐19 AND Botswana AND (intra action OR intra‐action)* and thereafter *COVID‐19 AND South AND Sudan AND (intra action OR intra‐action)*. Global Health and OVID Medline(R) databases returned no results for either keyword search on Botswana or South Sudan. A multi‐field search of the SCOPUS database returned 57 documents using the keyword *covid‐19 AND south AND sudan AND (intra action OR intra‐action)*. After a review of titles and abstracts for article relevance two were selected.^19^, ^20^ A multi‐field search of the SCOPUS database returned 57 documents using the keyword *covid‐19 AND botswana AND (intra action OR intra‐action)*. After a review of titles and abstracts for article relevance one was selected.^21^ Please see Table 2 for details, and for four additional articles identified through authors' further reading of the literature. Three gray literature documents were included from the list of references in the journal articles identified in the search and through the authors' engagement with the literature on health systems, learning, and preparedness.

**TABLE 2:** Notable examples of single loop learning in Nigeria.

Challenges 2016/17	Identified recommendations 2016/17	Best practice actions observed 2018
Inadequate logistics and supply chain management information systems at all levels of care	• The logistics and operational support working group should establish Inventory and Logistics Information Management System and support procurement process for medicines, consumable, and commodities for disease outbreak preparedness and response	• Availability of logistics (drugs, consumables and vehicles) reduced morbidity and mortality
Sample collection and transportation/need for standard laboratory support for prompt diagnosis and treatment	• NCDC should establish a national VHF sample transportation logistic framework by partnering with courier services with the provision of triple packing and develop the terms of reference (TOR) for partnership • NCDC should share protocol and policy for sample management with stakeholders and health facilities • All stakeholders should plan and preposition adequate PPE and other logistics to encourage prompt collection and handling of samples • Training and re‐training of appropriate health workers by NCDC and States.	• Development of National Testing Algorithm for Lassa fever improved the quality of test results and standardized laboratory procedures • Standardization of laboratory data template harmonized laboratory data • Capacity building of NRL staff by ISTH improved turnaround time in decision‐making for outbreak response
No isolation center	• Every health facility should have holding/isolation area for suspected cases. • Every state should establish at least one designated treatment center with a constituted case management team/IPC team for the management of Lassa fever and other VHFs	• Trained Lassa fever case management teams identified in the treatment centers reduced exposure of healthcare workers, which also reduced onward transmission to other patients and improved case detection.
No adherence to burial practices	• Risk communication team at the state level should sensitize community and train people of major faiths/religious on safe burial practices • Federal and states to conduct Advocacy to Government to enforce the implementation of existing public health policy that mandates environmental health officers linked with the DSNO to take charge of safe burial practices	• Identification of a facility‐based safe burial team (in Bauchi State) increased co‐operation from relatives and helped contain the spread of infection.

Abbreviations: DSNO, Disease Surveillance and Notification Officers; ISTH, Irrua Specialist Teaching Hospital; NCDC, National Centre for Disease Control; NRL, National Reference Laboratory; VHF, Viral Haemorrhagic Fever.

### 2.3 Data extraction and analysis

**FIGURE 2:**
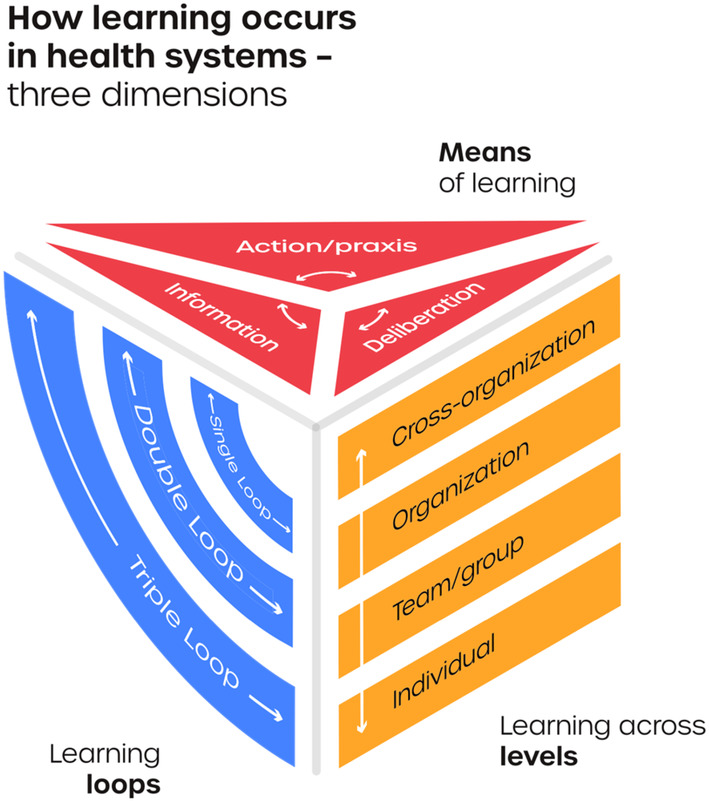
Three dimensions of learning in health systems Sheikh and Abimbola (2021).^4^

This conceptual analysis was conducted as a theory‐generating examination of the data from Action Reviews. The analysis utilizes the conceptual dimensions provided by the WHO's Alliance for Health Policy and Systems Research 2021 report on Learning Health Systems^4^—*means of learning*, *learning across levels*, and *learning loops* (as described below). See Figure 2.

**FIGURE 3:**
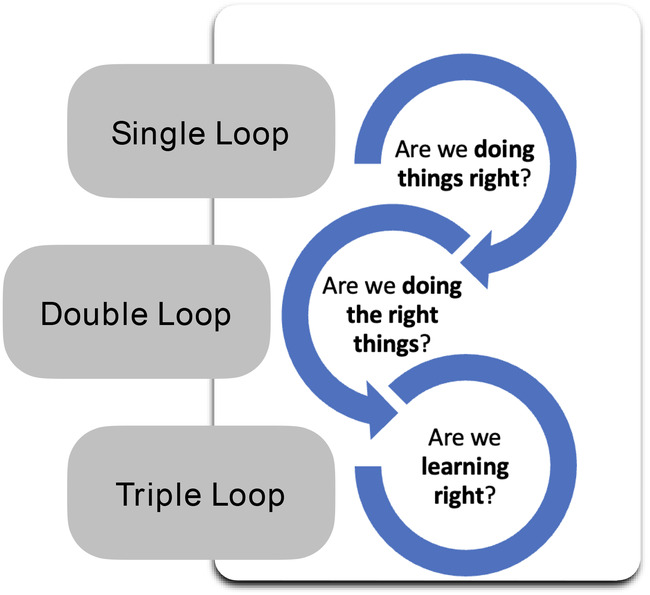
The three learning loops. Adapted from Sheikh and Abimbola (2021).^4^

The analysis began by reading closely through the paired Action Review reports, to identify notable examples learning, sorted into single loop learning, double loop learning, and triple loop learning^4^ as defined below (see Figure 3):

1. Examples of **single loop learning** are those that stem from or imply the question “are we doing things right?” They should demonstrate an effort to achieve the same goal but better—do more, try harder, new strategies. In the parlance of IAR and AAR they are minor course corrections.
2. Examples of **double loop learning** are those that stem from or imply the question “are we doing the right things.” They should demonstrate efforts are channeled to a different goal, which still addresses the problem but through another route. They often involve a change in goal/policy.
3. Examples of **triple loop learning** are those that stem from or reflect the question “are we learning right?” They demonstrate efforts to improve the ability to learn or change the current approach to learning, such as building new learning platforms, processes, linkages, infrastructure, and so on.

The second step in the analysis was to identify the conditions that enabled or constrained the notable examples of learning identified in the first step. We did so by referring to the paired Action Review reports and the academic and gray literature identified in each country for additional context. To understand the enablers and constraints on learning in each set of paired reports, we first identified if and how **learning across levels** occurred in each of the examples of learning—that is, whether learning had occurred at or been driven by or been driven across levels of actors or agents involved in learning: individuals, teams, organizations, or across different organizations (cross‐organizational). We also identified in each of the notable examples of learning, the **means of learning** involved, and which factors had enabled or constrained learning through the different means of learning—information, deliberation, and action—with definitions summarized by Millimouno et al^18^ as follows:

1. **Learning through information**: “[it] includes collecting, processing, deploying and disseminating information to meet the various learning aims of health systems, including measuring success and failure, anticipating trends and finding new approaches to address problems.”
2. **Learning through deliberation**: “[it] is about producing learning through acts of human deliberation. Processes of dialogue and reflection are essential to link past actions, the effectiveness of those actions, and future actions and consist of non‐peer and peer engagements and may occur in‐person or through technology‐enabled platforms.”
3. **Learning through action**: “[it] happens when people, whether individually or as part of a team, group or organisation, learn through the practice and iteration of tasks and projects.”

### 2.4 Country contexts

#### 2.4.1 Nigeria AAR Lassa Fever 2016–2018

As a federal republic with 36 states, Nigeria's constitution provides each state with its own budget and constitutional authority for health policy, including responding to health emergencies. The National Centre for Disease Control (NCDC) was first proposed in 2007 to integrate several relevant units of the Ministry of Health to respond to public health emergencies.

Outbreaks of Lassa Fever occur regularly in Nigeria. The 2018 Lassa Fever outbreak was the largest ever recorded, affecting 21 states in Nigeria, with 432 confirmed and 10 probable cases and 108 deaths (CFR 25%)—a CFR slightly lower than 2016/2017 of 28.7% of confirmed and probable cases.^22^, ^23^

An AAR, employing a working group format, was conducted after the 2016/2017 outbreak and again after the 2018 outbreak. Both included participants from federal and state ministries of health, NCDC, Lassa Fever steering committee meetings, physicians, and partners.^22^, ^23^ The six pillars selected for AAR in 2016/17 were (*Coordination; Case Management and IPC, Surveillance; Laboratory; Logistics; and Risk Communication)* were reorganized as five pillars in 2018 (*Coordination and Logistics; Case management, Safe Burial and IPC; Surveillance; Risk Communication and Social Mobilization; and Laboratory)*. The 2016/2017 AAR identified several challenges and recommendations. These have been linked to best practices and their stated impact identified in the 2018 AAR (see Supplementary Material S1).

#### 2.4.2 South Sudan IAR Covid‐19 2020–2021

Gaining independence from Sudan in 2011, South Sudan has been challenged by political, social, and economic stability and remains one of the world's most fragile states.^24^ It has decentralized health services with the national ministry of health providing guidance, funding, monitoring, and evaluation capacity for State, County, and Community levels in the country. South Sudan activated a Public Health Emergency Operations Centre (PHEOC) for COVID‐19 on February 3, 2020 and an incident management team to institute preparedness measures following the WHO's declaration of a PHEIC on January 30, 2020. The identification of the first COVID‐19 case on 5 April then established a full coordination framework, which included the COVID‐19 National Taskforce, National Steering Committee including eight Technical Working Groups, and the COVID‐19 State Taskforce Committees. As stated in both the 2020 and 2021 IAR:

“*The intent of the intra‐action review (IAR) is to assess the functional capacity of the public health and emergency response systems and to identify practical areas for immediate remediation or continued improvement of the current response to the COVID‐19 outbreak*.”^25^ *(South Sudan, 2020:3)*

Two pillars, *Surveillance, case investigation and contact tracing* and *National laboratory system*, were documented across the two IAR reports 2020 and 2021.^25^, ^26^ Their findings are identified in the tables found in Supplementary Material S2. *Vaccination* was added as a pillar in 2021 but did not appear in 2020, as such it was not included in this analysis as a separate pillar. However, to retain relevant data, where information appeared within the *Vaccination* pillar relevant to recommendations or challenges identified in 2020, it was included in the analysis under one of the two pillars. Due to the COVID‐19 emergency measures, participation was held both in person and online, to align with local social distancing requirements.

#### 2.4.3 Botswana IAR Covid‐19 2020–2021

Botswana, a parliamentary republic of 2.2 million people, is Africa's most stable country across political, social, and economic indicators and has a functional democracy.^24^ Botswana provides universal healthcare through a decentralized health system comprising 27 health districts. The country recorded its first COVID‐19 case on March 30, 2020. The objectives of the IAR 2021^28^ were set out as follows:

1. To provide an opportunity to share experiences and collectively analyze the ongoing in‐country COVID‐19 vaccination rollout, by identifying challenges and best practices.
2. To facilitate consensus building and compile the lessons learned by various stakeholders.
3. During the response to improve the current COVID‐19 vaccination rollout by sustaining best practices that have demonstrated success and by preventing recurrent errors.
4. To document and apply lessons learned from the COVID‐19 vaccination rollout efforts to date.

The Botswana IAR 2021 report was structured around a National Deployment and Vaccination Plan rather than specific pillars. As such for this analysis, relevant information, recommendations or challenges identified in the 2020 IAR, were included in the analysis under one pillar of *Vaccination*.^27^, ^28^

## 3 RESULTS

All the AARs and IARs included in this analysis demonstrated their ability to collect and process information and facilitate learning through human deliberation. They did this through participants identifying challenges, recommendations, and best practices; information which was then collected into reports. The final reports of the Action Reviews enabled the future deployment and dissemination of the information. The ability for this information to be used to find new approaches to solving problems depended on the creativity of participants involved in the AAR and IAR themselves and on the political will of senior leaders to employ such new approaches to problems and their solutions within relevant teams and organizations.

The inclusion of senior Ministry of Health (MOH) officials, other key stakeholders, and those closer to the ground (i.e., the policy implementers) in all AARs and IARs supported the problem‐solving that shows learning through information. Both learning through deliberation and learning through action were enabled by the design of the IARs and AARs working group methods. The reviews were, by design, platforms for deliberation. The realization of learning through action was apparent in the completion of actions (practice and iteration of tasks and projects) outside of those who participated in the AARs and IARs, thereby inferring wider team, organization, and health system learning. This outcome was also supported by the participation of senior leaders in the Action Reviews who were able to influence the uptake of learning across the relevant teams and organizations within the health system. Specific examples of single loop learning, double loop learning, and triple loop learning are identified below.

### 3.1 Single loop learning

#### 3.1.1 After‐action reviews

##### Nigeria

Positive feedback from participants was recorded during the Lassa Fever AAR in Nigeria, suggesting that individual participants were engaged in the process. Seventy percent of participants agreed that the AAR allowed participants to share experiences and identify challenges, gaps, and best practices encountered during the course of the response in 2018. This may be described as an endorsement of learning through information and deliberative learning, both of which support each of the three loops of learning, including the examples of single loop learning below (see Table 2). Table 2 displays some challenges identified in the 2016/17 report, subsequent recommendations from that year, and then matched with best practice actions cited as having been implemented in the following year's report. Notable examples of single loop learning in the AAR reports are highlighted in Table 2. The design of the AAR aims to surface knowledge including challenges, recommendations, and best practices, thus supporting single loop learning. Other conditions that may have facilitated single loop learning, include the seasonality of Lassa Fever incentivizing the implementation of lessons learned.

#### 3.1.2 Intra‐action reviews

##### South Sudan

The objectives of South Sudan's COVID‐19 IAR themselves demonstrate the means of learning that the review process aimed to achieve. Specific Objective 1 aimed “to share experiences” (i.e., gather information) and “collectively analyze by identifying challenges and best practices” (i.e., deliberate).

Learning across levels was identified in Specific Objective 2 “to facilitate consensus building and compiling of lessons learned by various stakeholders” (i.e., deliberate and gather information and learn from the action of others across the health system). Specific Objective 3 was to “document and apply lessons learned from the response to date to enable health system strengthening” (i.e., action).

Involvement of senior government officials, for example, the chairing of the COVID‐19 National Taskforce by the Vice President, and the involvement of technical agencies (e.g., WHO, UNICEF, MSF, and WFP) enabled cross‐organizational learning between national and international technical experts. COVID‐19 social distancing requirements meant that the IAR was conducted online in 2020 and 2021 allowing participation from WHO Headquarters and Regional Offices, further supporting cross‐organizational learning. The final report of the COVID‐19 IAR was prepared by the technical working group and provided to the COVID‐19 National Steering Committee which was then charged with integrating the recommendations into the National COVID‐19 Strategic and Response Plan.^25^

Completion of the IARs' three objectives supported the surfacing of challenges and recommendations in 2020 and the pairing of best practice actions in 2021 leading to single loop learning (see Table 3). Other conditions that supported single loop learning were the narrowing of the response pillar reviews from eight pillars in 2020 to three in 2021. This narrowing of the scope of the IAR to concentrate on issues of most importance allowed for more focused and responsive learning in 2021. This allowed for a greater depth of knowledge to be surfaced and targeted for correction in 2021. Moreover, the narrower scope for the 2021 IAR likely allowed for a more targeted selection of participants according to expertise—an inference that is apparently supported by the included attendance lists.

**TABLE 3:** Notable examples of single loop learning in South Sudan.

Challenges 2020	Identified recommendations 2020	Best practice actions observed 2021
Limited number of cases detected through the existing health system, healthcare facilities, surveillance system, and community surveillance system	• Deploy IDSR and Early Warning, Alert and Response System (EWARS) resources including surveillance focal points at a health facility; county; state; and national level (HF SFP; CSOs, SSOs, EPR department), RRTs, and EWARS electronic platform for the detection, reporting, investigation, and responding to COVID‐19 suspect and confirmed cases • Continue implementation of community‐based contact tracing as a model for engaging communities and improving compliance with contact listing and follow up • Activate community surveillance via Boma Health Initiative • Implement cross‐border surveillance planning	• Availability of surveillance system—Early Warning, Alert and Response System (EWARS) for Acute Respiratory Tract Infection (ARI) alert reporting and verification • Contact tracing formation and activation; teams are available to conduct contact listing and follow‐up • Availability of COVID‐19 guidelines, SOPs, and protocols which allowed the contact tracing team and RRT to timely response investigate and follow up on confirmed cases and their contacts
Inventory management is still weak with a need to recruit appropriately trained human resources, additional storage space; and software to manage laboratory test kits and other commodities for efficient and uninterrupted laboratory operations.	• Expand the National Public Health Laboratory (NPHL) space for sample and supplies storage, stock management	• Efficient stock management system; the NPHL did not experience stockout of reagents and other consumables
There was limited IPC compliance in the NPHL molecular laboratories as there was no IPC enforcement policy	• Develop laboratory IPC compliance protocols and enforce their consistent use in the NPHL molecular laboratory	• The National Public Health Laboratory (NPHL) monitored quality control testing in private laboratories

Abbreviations: IDSR, Integrated Disease Surveillance and Response; RRT, Rapid Response Team.

Notable examples of learning across levels leading to single loop learning in South Sudan are below.

##### Botswana

Several notable challenges and recommendations in 2020 can be seen reflected as Best Practices in the Vaccination pillar observed in 2021. They include responding directly to challenges and recommendations such as by clarifying roles and responsibilities and developing a communication strategy and implementation plan. The selection of only one pillar in 2021 concentrated the COVID‐19 IAR onto one specific area of focus enabling deeper learning. For this conceptual analysis, it limits the ability to view learning across other pillars. However, the focus on vaccination in the 2021 IAR, after having completed a comprehensive IAR in 2020, allowed a more focused participation of the MOH and the Botswana Medicines Regulatory Authority, as well as UN agencies.

There are examples of *learning through action* in the IAR of 2020 which led to single loop learning including the identification of the need for a clarification of roles and responsibilities (see Table 4). This and other notable examples below manifested, in the subsequent IAR of 2021, as best practices and what may be considered double loop learning—for example, clear organizational roles and responsibilities leading to the coordination of the vaccine rollout by the presidential task force and a Ministry of Health and Wellness (MOHW) Control Command under DHS facilitating the flow of information to lower levels of organization.

**TABLE 4:** Notable examples of single loop in Botswana.

Challenges 2020	Identified recommendations 2020	Best practice actions observed 2021
Delays in the development and dissemination of guidelines emanated from lack of role clarity on development and constant review of guidelines	• Clarification of the roles, terms of references (TORs), and responsibilities of the Public Health Emergency Management Committee (PHEMC), National Emergency Operation Centre (NEOC), Presidential Task Force	• The presidential task force coordinates the vaccine rollout while MOHW provides technical and operational support • There is an MOHW Control Command under the DHS office that facilitates the flow of information to the districts on the rollout
Lack of a Risk Communication and Community Engagement (RCCE) strategy before the response led to poor coordination of activities	• Review and disseminate the COVID‐19 RCCE strategy to the sub‐national level • Streamline and align the information management and flow between the RCCE Technical Team and MOHW top management • Enforce the Public Health Social Measures (PHSM) at the community level	• Development of Communication Strategy and Implementation Plan for vaccine acceptance and uptake • Undertaking vaccine acceptance rate and risk perception survey for the general public • Demand creation and uptake national campaign launch (#ArmReady) • District activation of demand creation and uptake campaign launch • Widely publicized vaccination activations on various media platforms

### 3.2 Double loop learning

Double loop learning stems from asking “are we doing the right things and thus manifests as efforts channeled to a different goal.” However, we found that in the Action Reviews, single loop learning (i.e., efforts to do things right) are often embedded within examples of double loop learning (i.e., efforts to do the right things). In such instances, while the goals may remain unchanged, the policy or strategy through which to achieve those goals may change significantly. This change in policy or strategy may then create new goals, alter existing goals, or impact beyond the initial goal.

#### 3.2.1 Intra‐action reviews

##### South Sudan

In South Sudan, given the 2020 challenge of low testing capacities and lab information management, COVID‐19 testing was decentralized to states (a change in “policy”), addressing the problem through bringing governance and testing itself closer to the ground. This was supported by further best practice actions that reflect single loop learning, such as improved training for laboratory staff, and monitoring through the National Public Health Laboratory (see Table 5). This learning was enabled by the diverse pillars explored in the first IAR and the broad expertise among participants. It may also have been further enabled by the sub‐optimal performance as measured by low per capita testing rates which in the early phase of the pandemic would have placed pressure on the health workforce.

**TABLE 5:** Notable examples of double loop learning in South Sudan.

Challenges 2020	Identified recommendations 2020	Best practice actions observed 2021
Low testing capacities at National Public Health Laboratory (NPHL) leading to sample backlog, and inadequate peripheral testing thus overall, the current per capita testing rate for the country remains below the optimal level of 10 tests per 10 000 population per week. The major limiting factors being inadequate utilization of the peripheral laboratories despite expansion of testing; manual sample extraction before PCR testing, nascent laboratory network with no private laboratory accredited to conduct molecular testing (GeneXpert or PCR)	• Establish NPHL owned Lab Information Management System to improve management of laboratory information and sharing with other programs and pillars including the PHEOC • Develop laboratory IPC compliance protocols and enforce their consistent use in the NPHL molecular laboratory • Expedite the Ag‐RDT verification and rollout to optimize the current country COVID‐19 testing capacities • Conduct regular laboratory operations meetings to promptly address emerging operational challenges	• COVID‐19 testing decentralized to the states through GeneXpert testing sites • The NPHL monitored quality control testing in private laboratories • Trained laboratory staff at all levels (national and sub‐national) with well‐structured modules and professional experts • Reviewed training materials with the surveillance team

##### Botswana

In Botswana, the 2020 COVID‐19 IAR report recommended the establishment of key institutions, such as a fast‐tracked fully functional National EOC. The National EOC was established by 2021. The establishment of the National EOC with district‐level representatives addressed an identified need for better operational structures for surveillance and management at different levels of governance. In doing so, the goals remain unchanged but the local structures to achieve these goals were bolstered. Elsewhere, the identified lack of a communication plan was addressed by not just a communication plan (as in previous single loop learning) but the formation of a community of observers and monitors and the involvement of religious and traditional leaders in communication activities. Enabling these learnings were the multi‐pillar COVID‐19 IAR in 2020 that allowed for a broad evaluation of gaps in the health system and recommendations for correction. This was supported by the wide range of actors with different expertise and backgrounds that participated in the first IAR, and likely propelled by the pressure on health governance and its actors during the early stages of the pandemic (Table 6).

**TABLE 6:** Notable examples of double loop learning in Botswana.

Challenges 2020	Identified recommendations 2020	Best practice actions observed 2021
Delays in the development and dissemination of guidelines emanated from a lack of role clarity on development and constant review of guidelines	• Enhance the capacity of the emergency preparedness and response teams at national and subnational level • Train the Public Health Emergency Management Committee (PHEMC) on Incident Management Structures and EOC roles and functions	• Established National Emergency Operation Centre (NEOC) with representative structures at the district level (DEOC) which provide support on operational and tactical issues, e.g., pooling transport from different departments to support the rollout • Appointed liaison officers for all the districts as a constant communication and technical link between national and implementing districts
Lack of/inadequate Communication Plan for community	• LGA health team to establish a social mobilization working group committee responsible for risk communication at the LGA level • State educators to provide regular content/activity reviews on their Lassa fever programs with the National working group	• Formation of community observers and monitors to ensure adherence to positive food handling and environmental sanitation led to community ownership and sustainability • Involvement of religious and traditional leaders in sensitization activities increased grassroots awareness, allowed prompt dissemination of information and supported debunking rumor and misinformation

Abbreviation: LGA, Local Government Area.

### 3.3 Triple loop learning

Examples of triple loop learning were less common in the Action Reviews included in this analysis. No clear examples of triple loop learning were present among the COVID‐19 IARs in South Sudan. In Botswana, the creation of the National EOC identified in the 2020/21 IAR, an example of double loop learning, supported the launch of the Botswana Public Health Institute in 2022. This may be viewed as an example of triple loop learning as it developed as a new structure that engaged with new actors and embarked on new learning. This may suggest, like previous learning, that double loop learning is embedded in triple loop learning providing a catalyzing process for assessing “are we doing the right things” and accelerating questions of “are we learning right.”

One of the clearest demonstrations of triple loop learning was evident in the AARs in Nigeria (see Table 7). The formal establishment of the NCDC through an Executive Bill in 2018 addressed an identified 2016/17 challenge of potential legislative obstacles and bureaucracy and supported the recommendation to streamline the legislative processes for funding, promoting risk communication and advocacy to lawmakers. Under the Bill, the role of the NCDC was formalized and its mandate as a federal agency was established. The NCDC provides technical and logistical guidance to states and local governments for “planning, implementation and management of diseases of public health importance and on activities to reduce health risk and impact from public health events.”^29^ As such the NCDC has specific convening power and technical expertise to bring together states for future AAR and to institute a roadmap to implement state or federal‐level recommendations. As an example of triple loop learning, the NCDC therefore helps to answer the question “are we learning right.” The regular occurrence of disease outbreaks in Nigeria creates the need for real‐time learning and responsive governance^16^ and NCDC convenes an annual critical review meeting of epidemiologists to review recent public health outbreaks and events.^12^ The regular occurrence of the review meeting provides the conditions to engage with the same actors each year and formalize pathways to convert AAR recommendations into policy.

**TABLE 7:** Notable examples of triple loop learning in Nigeria.

Challenges 2016/17	Identified recommendations 2016/17	Best practice actions observed 2018
Legislative obstacles and bureaucracy	• Advocate to lawmakers to streamline the legislative processes for funding and promoting risk communication activities • Personalizing message content to leaders	• Involvement of highly placed government and political officials as champions for Lassa fever communication allowed the release of funds for sensitization in LGAs, increased awareness, and acceptance • Collaboration between States, NGOs, and partners to reach a larger community on Lassa Fever awareness and sensitization led to increased awareness, positive behavioral change • Formation of community observers and monitors to ensure adherence to positive food handling and environmental sanitation led to community ownership and sustainability

Abbreviation: LGA, Local Government Area.

## 4 DISCUSSION

The design of both the COVID‐19 IAR and the AAR were successful in surfacing knowledge and, by engaging the right (in different contexts) actors in the reviews, asking “are we doing things right.” As such, and unsurprisingly given it is the simplest form of learning demanding minor course correction, single loop learning was evident in all the reports. Moreover, we found that single loop learning is often embedded within examples of double loop learning. This provided a more transformative basis for policy change and assessment of “are we doing the right things.” Triple loop learning was evident in AAR, and less in COVID‐19 IAR in Botswana. Single loop learning being embedded in double loop learning, and double loop learning being embedded in triple loop learning suggests a *catalyzing effect* of the learning loops. Put plainly, once the learning journey has begun—even in the simplest of single loop learning—a “habit” is formed that is geared toward deeper learning. This stepped approach may provide the groundswell and evidence base for new policy or accelerating institution development—such as in the case of the Botswana Public Health Institute or the formal establishment in law of the NCDC. Regarding the distribution, single loop learning was the most common and triple loop learning was the least common.

Several conditions were identified that may have supported learning. First, the expert and relevant knowledge of participants, including the political buy‐in from senior officials seen through their presence in publicly supporting the Action Reviews. Second, the iterative process of COVID‐19 IARs that were initially wide in aperture covering various pillars to be focused in their second iteration on specific pillars. This was similarly supported by relevant tailoring of participant lists. This finding provides some validation for one of the potential mechanisms for the effectiveness of Action Reviews set out in WHO's Guidance for AARs^8^: *Its success hinges on the ability to bring relevant response stakeholders together in an environment where they can analyze actions taken during the response in a critical and systematic fashion and identify areas for improvement*.

A further condition that potentially supported learning was the presence of international organizations and NGOs. This may have supported the cross‐organizational learning during COVID‐19 IARs, with particular relevance given the wide impact of the pandemic and the potential for cross‐border learning. However, little is relayed in the reports about what was discussed by these participants. Other learning across levels is similarly hard to assess without an embedded study (with researchers being part of the Action Reviews to study and document the process), but we assume there were important interactions between different levels of seniority, different teams with MOH and more broadly, and between individuals. The quantity and quality of these interactions are not recorded in the reports but may have had a lasting impact on health system learning, including in preparedness, response, and resilience.

For optimal learning, maintaining the same participants across several years may support the higher achievement of triple loop learning and deep learning outcomes. This may however be unhelpful where a deep dive into specific capabilities or subject is required. Nevertheless, as demonstrated in South Sudan's paired reviews, maintaining some of the original cohorts across different years may support the translation of lessons learned enabling multi‐year learning processes. Moreover, this may support the development of *champions of change* with a deeper understanding of the process and identified changes to translate into deeper organizational and system transformation.

The WHO's Integrated Disease Surveillance and Response Technical Guidelines recommend the establishment of Public Health Emergency Management Committees at state and local levels. The establishment of these Committees aims to support the implementation of recommendations, such as those produced during Action Reviews. However, in the case of Nigeria these were found to often be “non‐existent or partially constituted” diminishing the implementation of recommendations of AARs.^12^ This surfaces one of the key weaknesses with such methods of learning—recommendations are often not fully implemented. The repetition of such methods over time, as demonstrated in this analysis, may improve the implementation of recommendations.

Implementation of recommendations from review processes is one metric to evaluate that impactful learning has taken place. When learning platforms like Action Reviews do not lead to action, the cost–benefit for organizations to conduct such processes is significantly reduced. Detecting, notifying, and responding to infectious disease outbreaks early can significantly curtail their spread—recent advocacy and WHO Guidelines on Early Action Review^10^, ^15^ promote the benefits of timely intervention. In South Sudan, not all recommendations made in the 2020 COVID‐19 IAR were implemented in a timely manner.^20^ Recommendations to address poor knowledge and stigma around vaccination, leading to vaccine hesitancy, were not fully implemented as of 2022.^20^ This is a common criticism of AARs, and further emphasis on developing policy pathways may be incorporated into Action Reviews—as has been done in the new WHO Guidance for the conduct of COVID‐19 AAR.^30^ This raises the need to change the current approach to ensure the implementation of recommendations from Action Reviews.

All the means of learning were on display. The collection and processing of information was evident and demonstrated across the years in all three countries. These were supported by deliberation during the Action Reviews which produced challenges and recommendations. The collaborative approach used during the Action Reviews demonstrates action or praxis between different learning levels, which may have iteratively improved the quality of the Action Reviews. All three occurred simultaneously.

IARs and AARs are inherently different and aimed at providing learning at points in the emergency cycle. IARs aim to course correct during a health emergency, while AARs are designed for deeper learning after a health emergency. COVID‐19 IARs attempted to fine‐tune health system responses during an emergency—that is, largely single loop learning with the potential for double loop learning. AARs are more targeted at uncovering deeper critical and corrective behaviors that may lead to changes in policies, governing norms, or realigning objectives—that is, largely double loop learning with potential for triple loop learning. The lack of examples in this study of triple loop learning during COVID‐19 IARs may be due to the IARs being designed to capture and identify short‐term course correction during a health emergency rather than geared toward transformational change. The novelty and the depth and breadth of impact of the COVID‐19 outbreak across the respective countries may also have affected the two countries' ability to achieve triple loop learning.

The full continuum of Action Reviews and other preparedness and response activities should support system‐wide “learning how to learn” or triple loop learning and double loop learning that challenges assumptions of the health system and its function—outside of periods of public health emergencies to prepare health systems to better learn and respond. Future research may explore this full continuum of Action Reviews, including the Guidance for the conduct of country COVID‐19 AAR, and their impact on both triple loop and double loop learning.

The three paired Action Reviews in this conceptual analysis demonstrate the utility of such reviews both after and during a public health emergency or event. Action Reviews are not, however, an end in themselves but rather a tool to support health system learning and strengthening. This conceptual learning analysis found that health information management and disease surveillance, health system coordination, and workforce development were three areas on which the Action Reviews appear to have impacted positively. They repeatedly emerged as areas where information was uncovered on specific or systemic issues and improvement was reported over the course of the paired reviews.

While the participant surveys are important to understand learning by individuals, and to some extent teams or groups, they are insufficient to assess learning at the organization and cross‐organization levels which are required for deeper and lasting preparedness, response, and resilience. As such the examination of learning within one specific disease within the same country and across multiple years may be informative for deeper learning at team/group and organization levels. The full participant list was not publicly available for Nigeria or Botswana. In South Sudan, of those who signed attendance sheets across the 2 years, few were present for both the 2020 and 2021 IARs. As such, assessing the depth of *individual learning* is more difficult. The different makeup of participants means that individual learning across both years would have been limited and *triple loop learning* harder to obtain. This is however unsurprising given the more targeted focus on vaccination in 2021.

This study built on existing frameworks for analyzing health system learning.^4^, ^5^, ^6^ It also builds on earlier analyses of the impact of action reviews.^12^, ^13^, ^14^, ^18^, ^31^, ^32^, ^33^, ^34^ Unlike previous work in this field, this study brings together earlier work on AAR and recent data on COVID‐19 IAR, using a new and robust conceptual framework for learning health systems.^4^ The same framework was used by Millimouno et al^18^ to analyze health system learning in Guinea in the context of multiple disease outbreaks—Ebola virus disease, Measles, Lassa Fever, COVID‐19, and Marburg virus disease. Building on Millimouno et al's study design^18^—a retrospective longitudinal single embedded case study design—our study included three paired cases in Nigeria, Botswana, and South Sudan, with data from respective AAR and IAR reports, academic literature, and gray literature. Our theoretical proposition was the same as Millimouno et al's (2023)^18^: that a learning health system (or platform) must include all the learning dimensions (loops, means, and levels). However, a unique strength of our study is that it utilized paired reports of documented learning and employed a systematic approach that may be used as an example for further studies to analyze learning, improve learning outcomes, generate more robust policy development, and provide insights into how health systems can learn better.

A limitation of this analysis is that we relied on what was described in the report which constrains a fuller explanation of what is really going on in each example of learning. For example, the contextual factors that enabled or constrained learning could only be inferred from the reports and literature included in this analysis. As such, this study could only establish potential and conceptual connections between learning and health system outcomes; other contextual factors beyond learning play an important role in generating the actions that signify that learning has taken place. Similarly, different cultures and country contexts may impact on how learning may be reported or when specific learning outcomes may be identified or achieved. Future studies with an embedded design—with researchers observing and documenting events during and after the reviews—may further qualify and enrich these links. Another limitation of this study is the paucity of paired case studies—only those able to be paired and publicly available on WHO's Strategic Partnership for Health Security (SPH) website were included. The inclusion of further paired studies would strengthen the findings and conclusions of the analysis. To do so, future action reviews should be made publicly available by MOHs and through WHO to allow such paired analysis.

Future Action Reviews may consider employing an embedded research approach such that the report is prepared by researchers embedded within the Action Review itself, allowing for the possibility of generating richer accounts of learning within and across levels, and of the conditions that enabled or constrained such learning. Similarly, future reports of Action Reviews may consider framing learning explicitly in single, double, or triple loop learning, while also providing greater detail on how learning occurred. Additionally, future reports of Action Reviews may consider including both qualitative and quantitative components—such as participant surveys and structured interview questions eliciting examples of different loops of learning, means of learning, and levels of learning, for Action Reviews of reoccurring outbreaks, time‐bound epidemiological data. The inclusion of the same participants over two or more consecutive Action Reviews may provide for learning that builds on past Action Reviews and continuity of lessons learned from previous health emergencies.

As George Bernard Shaw said, “if you teach a man anything, he will never learn it.” The inclusive and catalytic approach of the IARs and AARs, by involving knowledge holders including on‐the‐ground policy practitioners, allows ownership over policymaking to improve health emergency preparedness, response, and resilience. It allows responders, practitioners, and policymakers to climb together into the driver's seat and build better policy. Future studies should attempt to identify how such policy development can be further optimized for health emergencies and for the health system more broadly.

## 5 CONCLUSION

Action Reviews provide an opportunity for more informed health system governance practices and overall, more aware, critical, and corrective practices and routines. In turn, this has the potential benefit of improved performance of functions and increased adaptivity and innovation. However, double loop and triple loop learning is underutilized. It should be utilized more for transformational change and increased ability for continuous learning. This may be achieved by encouraging participants of past AAR and IAR into current Action Reviews to enable continuous learning and ensure that relevant influential actors within the health system participate. This conceptual learning analysis demonstrates that Action Reviews, by design, surface knowledge. When properly conducted according to WHO Guidelines they can support the codification of knowledge. With proper follow through this knowledge can then be applied and lead to corrective and innovative actions to improve health system performance, and in exceptional cases lead to transformational change and continuous learning.

## FUNDING INFORMATION

No funding was sought or received to complete this study.

## CONFLICT OF INTEREST STATEMENT

This work was carried out as part of Elliot Brennan's PhD research at the University of Sydney. It does not represent the views of any past affiliation with the WHO or other organizations. Elliot Brennan is a PhD candidate at the University of Sydney. He has worked as a consultant for the World Health Organization's Country Simulation and Review Unit in Geneva and the WHO Western Pacific Regional Office Strategic Dialogues Unit. He has designed and facilitated COVID‐19 after action reviews, simulation exercises, and other strategic dialogs with the aim of system learning during and after crises. Seye Abimbola is a health systems researcher from Nigeria. He is currently based at the University of Sydney in Australia, where his teaching and research focus on knowledge practices in global health, health system governance, and the adoption and scale up of health system innovations. Dr Abimbola was awarded the 2020–2022 Prince Claus Chair in Equity and Development at Utrecht University in the Netherlands for his work on justice in global health research. He is the editor‐in‐chief of BMJ Global Health.

## Supporting information


**Data S1.** Supporting Information.
