# Supplementary material for: A conceptual learning analysis of paired after action and intra action reviews for health emergencies
Source: Learn Health Syst. 2024 Aug 29;8(4):e10447. doi: 10.1002/lrh2.10447 (PMC11493552; doi:10.1002/lrh2.10447)
Supplement: Supplementary file 1 — Data S1. Supporting Information. [file LRH2-8-e10447-s001.docx]

## Supplementary Material: A Conceptual Learning Analysis of Paired After-Action and Intra-Action Reviews

### Supplementary Material 1. Results Nigeria AAR 2016/17 to 2018

**Coordination and Logistics**

| **Challenges 2016/2017** | **Identified Recommendations 2016/2017^[[1]](#footnote-1)^** | **Best Practice Actions Observed 2018^[[2]](#footnote-2)^** |
| --- | --- | --- |
| Weak political support | - Federal Ministry of Health/NCDC to conduct high level advocacy to political stakeholders (Governor’s forum, NCH, National Task Force meetings etc.) | - Political will and commitment including government, partner and media support, was identified as an overarching enabling factor |
| Resource mobilization / funding | - States and LGAs to establish rapid response team (RRT) with appropriate composition of expertise | - Availability of logistics (drugs, consumables, vehicles) reduced morbidity and mortality - Availability of funds at National level, and some states, allowed timely intervention and prepositioning of resources |
| Inadequate outbreak preparedness | - Conduct risk assessment to inform preparedness plan and enhance advocacy for funding - States to establish multi-hazard EOCs and incident management system for coordination of outbreak response - States should develop multi-hazard preparedness and response plan | - Multi-sectoral collaboration allowed a comprehensive and harmonised response combined supported by improved capacity of workforce, resources, funding and political will |
| Low motivation of Health workers in outbreak response | - Health workers should be motivated through incentives such as training opportunities, promotions, special commendations | - Capacity building allowed a skilled workforce that was confident and competent |
| Lack of cohesive logistics Technical Working Group (TWG) | - States to integrate Logistics and Operational Support Working Group into Emergency Preparedness and Response (EPR) team - Director of Public Heath, Director of pharmaceutical services and State Epidemiologist | - Formation and reactivation of EOCs |
| Inadequate logistics and supply chain management information systems at all  levels of care. | - The logistics and operational support working group should establish Inventory and Logistics Information Management System and support procurement process for medicines, consumable and commodities for disease outbreak preparedness and response | - Availability of logistics (drugs, consumables, vehicles) reduced morbidity and mortality |
| Insufficient needs assessment undertaken to develop a contingency plan | - Carry out a Logistics needs assessment and forecasting by RRT members in every state - States supported by NCDC to train and retrain RRT, LGA teams and Health Facility teams on inventory management and supplies for outbreak responses - States should develop a contingency plan with business continuity plan | - Availability of funds at National level, and some states, allowed timely intervention and prepositioning of resources - Availability of logistics (drugs, consumables, vehicles) reduced morbidity and mortality |
| Insufficient and substandard PPEs |  | - Availability of logistics (drugs, consumables, vehicles) reduced morbidity and mortality |
| Insufficient number of dedicated vehicles for distributing medicines, supplies and  commodities from state stores to health facilities and end users in the community |  | - Availability of logistics (drugs, consumables, vehicles) reduced morbidity and mortality |
| State over dependence on the Federal Government and International Partners’  medicines and health commodities required for outbreak response | - States should dedicate funds for procurement of equipment, infrastructures, materials, medicines, consumables and vehicles for outbreak response | - Availability of funds at National level, and some states, allowed timely intervention and prepositioning of resources - Political will and commitment including government, partner and media support, was identified as an overarching enabling factor |

Prioritised Activities 2018 AAR: Coordination and Logistics

1. Generate a costed Lassa fever preparedness plan

2. Mobilize resources and technical support from partners for Lassa fever preparedness and response

3. Conduct monthly coordination meetings with Lassa fever stakeholders in states and Local Government Areas

**Surveillance**

| **Identified Challenges 2016/2017^[[3]](#footnote-3)^** | **Identified Recommendations 2016/2017^[[4]](#footnote-4)^** | **Best Practice Actions Observed 2018^[[5]](#footnote-5)^** |
| --- | --- | --- |
| Case reporting: Late/Under reporting | - Designate and train Surveillance focal persons in all health facilities, including tertiary and private facilities and community involvement in disease surveillance by states and LGAs | - Enhanced community-based surveillance allowed early detection and reporting and timely reporting of cases |
| Lack of validation and harmonization of data | - Regular review meetings and collaboration between all stakeholders at all levels involved in data management | - Surveillance focal persons in health facilities allowed timely reporting of cases - Deployment of SORMAS to some states allowed timely update of information - Use of other established system for surveillance integration enabled by human resources and partner support |
| Under-diagnosis of cases |  | - Surveillance focal persons in health facilities allowed timely reporting of cases - Functional RRT in states allowed a timely response - Deployment of SORMAS to some states allowed timely update of information |
| Poor/non conclusive outbreak investigation | - Provision of budgetary allocation and timely release of funds for surveillance activities and training at all levels | - Functional RRT in states allowed a timely response - Deployment of SORMAS to some states allowed timely update of information |
| Poor coordinated outbreak response | - NCDC to provide guideline for incident management system to States - NCDC to provide regular oversight function of the incident management system for States - Health facilities should follow the appropriate channel of communication | - Distribution of case definition in health facilities and communities allowed early detection of cases - Functional RRT in states allowed a timely response - Deployment of SORMAS to some states allowed timely update of information |
| Non-utilisation of data for decision making |  | - Surveillance focal persons in health facilities allowed timely reporting of cases - Deployment of SORMAS to some states allowed timely update of information - Use of other established system for surveillance integration enabled by human resources and partner support |
| Poor containment of outbreak | - Monthly review meetings of clinicians and surveillance team should be conducted in the State - States supported by NCDC to strengthen capacity of focal persons at health facilities and LGA on data management - States to strengthen capacity of surveillance team on active case search, contact tracing and monitoring | - Distribution of case definition in health facilities and communities allowed early detection of cases - Functional RRT in states allowed a timely response - Deployment of SORMAS to some states allowed timely update of information |

Prioritised Activities 2018 AAR: Surveillance

1. Designate surveillance focal person in all health facilities and community

2. Conduct IDSR training for all levels of the surveillance system

3. Mapping and sensitization of stakeholders and advocacy visit to them

**Laboratory**

| **Identified Challenges 2016/2017^[[6]](#footnote-6)^** | **Identified Recommendations 2016/2017^[[7]](#footnote-7)^** | **Best Practice Actions Observed 2018^[[8]](#footnote-8)^** |
| --- | --- | --- |
| Sample collection and transportation/Need for standard laboratory support for prompt diagnosis and treatment | - NCDC should establish a national VHF sample transportation logistic framework by partnering with courier services with provision of triple packing and develop the terms of reference (TOR) for partnership - NCDC should share protocol and policy for sample management with stakeholders and health facilities - All stakeholders should plan and preposition adequate PPE and other logistics to encourage prompt collection and handling of samples - Training and re-training of appropriate health workers by NCDC and States. | - Development of National Testing Algorithm for Lassa fever improved quality of test result and standardised laboratory procedures - Standardization of laboratory data template harmonized laboratory data - Capacity building of NRL staff by ISTH improved turnaround time in decision making for outbreak response |
| Sample Processing/timeliness of results | - NCDC to liaise with State government and re-activate non-functioning VHF laboratories with reagents, consumables, equipment, training and re-training of personnel - NCDC to establish a sustainable supply chain system for reagents and consumables to all the VHF laboratories - NCDC to develop and standardize a uniform testing platform for equipment, reagents and consumable across the VHF testing laboratories (for Quality Control and Quality Assurance purpose) - NCDC to establish at least one functional VHF laboratory in each geo-political zone - NCDC to develop a plan for equipment maintenance and replacement; contract with manufacturers and/or bio- medical engineers - NCDC to consider including private laboratories with capacity for VHF diagnosis in the network - NCDC to provide backup power system across VHF testing laboratories - States and other stakeholders (management of testing laboratory institution) provide adequate space and freezers for sample and reagent storage - Laboratory personnel should be motivated to provide essential services round the clock by management of testing laboratories | - Capacity building of NRL staff by ISTH improved turnaround time in decision making for outbreak response - NCDC-TRANEX sample transportation Mechanism allowed early delivery of samples to testing Laboratories - Mapping of Testing Laboratories increased the proximity of states to testing laboratories, improved efficiency of transportation of samples by courier company and reduced workload at ISTH |
| Proper sample collection and  Transportation | - NCDC to provide standard autoclaves, biohazard bags, bio-containment equipment and dedicated VHF sample storage freezers - Testing Laboratory institutions to provide adequate biosecurity and biosafety measures and delineate work flow path - Management of testing laboratories/NCDC to promote strict compliance to all IPC policies and guidelines | - NCDC-TRANEX sample transportation Mechanism allowed early delivery of samples to testing Laboratories - Mapping of Testing Laboratories increased the proximity of states to testing laboratories, improved efficiency of transportation of samples by courier company and reduced workload at ISTH. - Development of National Testing Algorithm for Lassa fever improved quality of test result and standardised laboratory procedures - Standardization of laboratory data template harmonized laboratory data |
| Provision of standard laboratories for Lassa fever diagnosis | - NCDC to support capacity building of personnel on laboratory inventory management in VHF laboratories - Testing Laboratory institutions to provide adequate budget line for reagents and consumables - Testing Laboratory institution management to ensure procurement specification (equipment, reagents and consumables) | - Mapping of Testing Laboratories increased the proximity of states to testing laboratories, improved efficiency of transportation of samples by courier company and reduced workload at ISTH |
| Laboratory inventory | - NCDC to support capacity building of personnel on laboratory inventory management in VHF laboratories - Testing Laboratory institutions to provide adequate budget line for reagents and consumables - Testing Laboratory institution management to ensure procurement specification (equipment, reagents and consumables) | - Development of National Testing Algorithm for Lassa fever improved quality of test result and standardised laboratory procedures - Standardization of laboratory data template harmonized laboratory data |

*Prioritised Activities 2018 AAR: Laboratory*

*1. Training on data management for Medical Laboratory Scientists and all NCDC network*

*laboratories*

*2. Training and dissemination of SOPs on a sample management in all states*

**Case Management and IPC**

| **Challenges 2016/2017^[[9]](#footnote-9)^** | **Identified Recommendations 2016/2017^[[10]](#footnote-10)^** | **Best Practice Actions Observed 2018^[[11]](#footnote-11)^** |
| --- | --- | --- |
| No isolation centre | - Every health facility should have holding/isolation area for suspected cases. - Every state should establish at least one designated treatment Centre with a constituted case management team/IPC team for management of Lassa fever and other VHFs - NCDC to establish, equip, and maintain zonal referral centers that have the capacity to serve cluster of states for management of complicated Lassa fever and other VHFs cases - Implement dedicated budget line at facility, LGA, state, and national level for management of cases of Lassa fever and other VHFs | - Trained Lassa fever case management teams identified in the treatment centres reduced exposure of health care workers, which also reduced onward transmission to other patients and improved case detection. - Motivation of Lassa fever case management teams in form of stipends (in Kogi state) - ICU support for critically ill patients improved patient outcomes |
| Delay getting results | - NCDC to establish, equip and maintain more diagnostic laboratories across the country - NCDC to develop standard sample collection protocols to be used in holding areas, treatment centers and referral hospitals - State should be responsible for the transportation of samples to the designated laboratories - Basic PPEs and sample transportation kits should be prepositioned in all states and treatment centers, initial stock by NCDC and subsequent stocks by states - NCDC to develop protocol that guides real time release of laboratory result and identified routes of result dissemination and sharing. - Clinicians advised to also use other supportive clinical diagnosis such as urinalysis, FBC, AST and ALT for case detection | - Synergy between state-owned and federal institutions (in Taraba state) reduced loss to follow up, community transmission, and CRF due to early case detection. - Trained Lassa fever case management teams identified in the treatment centres reduced exposure of health care workers, which also reduced onward transmission to other patients and improved case detection. |
| Decisions being made based on clinical experience | - FMOH/NCDC to prioritize and facilitate research on case management, manufacturing of drugs, RDT kit and vaccine development | - Trained Lassa fever case management teams identified in the treatment centres reduced exposure of health care workers, which also reduced onward transmission to other patients and improved case detection. - Citing of the laboratory services for supportive investigations in the   isolation/treatment centres led to faster clinical decisions, reduced exposure for lab staff, and better management of patients |
| Late Detection |  | - Trained Lassa fever case management teams identified in the treatment centres reduced exposure of health care workers, which also reduced onward transmission to other patients and improved case detection. - Synergy between state-owned and federal institutions (in Taraba state) reduced loss to follow up, community transmission, and CRF due to early case detection |
| No adherence to burial practices | - Risk communication team at state level should sensitize community and train people of major faiths/religious on safe burial practices - Federal and states to conduct Advocacy to Government to enforce the implementation of existing public health policy that mandates environmental health officers linked with the DSNO to take charge of safe burial practices - NCDC to disseminate national guidelines to IPC to all states, LGAs and health facilities - NCDC in collaboration with Ministry of Environment should develop and distribute protocol to address waste management practices in health facilities/treatment centres | - Identification of a facility-based safe burial team (in Bauchi State) increased co-operation from relatives and helped contain the spread of infection. |

Prioritised Activities 2018 AAR: Case Management, Safe Burial and IPC:

1. Equip three treatment centres with ICU equipment to manage critically ill Lassa fever patients

2. Identify and train relevant HCWs in treatment centres on ICU care for Lassa fever

3. Identify and train IPC team/committee in the treatment centres

**Risk Communications and Social Mobilisation**

| **Challenges 2016/2017^[[12]](#footnote-12)^** | **Identified Recommendations 2016/2017^[[13]](#footnote-13)^** | **Best Practice Actions Observed 2018^[[14]](#footnote-14)^** |
| --- | --- | --- |
| Fear in Health workers (inadequate knowledge of IPC materials and strategies) | - States to lead sensitization of healthcare workers on universal precautions through adequate training that provide hands-on experience, as well as adequate provision of resources for universal precaution in health facilities - SOPs on universal precautions should be placed in strategic locations within health facilities by Treatment Centre Management Team |  |
| Inadequate/ gaps in health workers knowledge of Lassa fever and insufficient hands-on-experience | - States supported by NCDC to train of healthcare workers on how to appropriately wear and remove PPEs. - Each Treatment Centre should be supported by the SMOH to have an IPC committee to ensure best practices in IPC. |  |
| Inadequate IEC materials for health workers | - States to identify and engage with partners and social agents to develop comprehensive IEC materials | - Collaboration between States, NGOs, and partners to reach larger community on Lassa Fever awareness and sensitization led to increase awareness, positive behavioural change - Involvement of religious and traditional leaders in sensitization activities increased grassroots awareness, allowed prompt dissemination of information and supported debunking rumour and misinformation - Translation of jingles and IEC materials to local languages for effective reach increased acceptance of messages |
| Lack/inadequate of communication Plan for community | - The state epidemiologist should identify and meet relevant stakeholders to form a committee to develop a risk communication plan. (e.g. MDAs, health educators, etc.) in the community for effective risk communications. - LGA health team to establish a social mobilization working group committee responsible for risk communication at the L.G.A level. - State educators to provide regular content/activity review on their Lassa fever programs with the National working group | - Collaboration between States, NGOs, and partners to reach larger community on Lassa Fever awareness and sensitization led to increase awareness, positive behavioural change - Formation of community observers and monitors to ensure adherence to positive food handling and environmental sanitation led to community ownership and sustainability - Involvement of religious and traditional leaders in sensitization activities increased grassroots awareness, allowed prompt dissemination of information and supported debunking rumour and misinformation |
| Poor interface between the community healthcare worker and the community | - State epidemiologist to coordinate meetings to improve synergy between health educators, healthcare workers and community leaders to establish appropriate messages to be communicated to the community. - States and LGAs to sensitize the community to report every case of fever to the health facility | - Collaboration between States, NGOs, and partners to reach larger community on Lassa Fever awareness and sensitization led to increase awareness, positive behavioural change - Formation of community observers and monitors to ensure adherence to positive food handling and environmental sanitation led to community ownership and sustainability - Involvement of religious and traditional leaders in sensitization activities increased grassroots awareness, allowed prompt dissemination of information and supported debunking rumour and misinformation |
| Resistance/Denial by the community | - NCDC and States to develop multi-hazard communication approach to reduce resistance/denial of massages, this should include community leaders at all levels. - Balanced health messages that provide hope and reduce fear. | - Collaboration between States, NGOs, and partners to reach larger community on Lassa Fever awareness and sensitization led to increase awareness, positive behavioural change - Formation of community observers and monitors to ensure adherence to positive food handling and environmental sanitation led to community ownership and sustainability |
| Low or insufficient government commitment | - High level advocacy to the government/leadership of the state (including class A traditional ruler in a state, NCDC, FMOH initiated by stakeholders in infectious diseases in the state, Nigerian Governors Forum) | - Involvement of religious and traditional leaders in sensitization activities increased grassroots awareness, allowed prompt dissemination of information and supported debunking rumour and misinformation |
| Lack of strong advocacy to the leadership by health workers | - Using evidence based (fact sheets, data generated from within and photographs) to advocate to the leadership | - Collaboration between States, NGOs, and partners to reach larger community on Lassa Fever awareness and sensitization led to increase awareness, positive behavioural change |
| Inadequate funding for communication activities | - Advocate to Government to ensure release of budget earmarked for risk communication activities - Social mobilization working group to leverage on partners’ support and other influential personalities | - Involvement of highly placed government and political officials as champions for Lassa fever communication allowed release of funds for sensitization in LGAs, increased awareness and acceptance |
| Legislative obstacles and bureaucracy | - Advocate to lawmakers to streamline the legislative processes for funding and promoting risk communication activities. - Personalizing message content to leaders | - Involvement of highly placed government and political officials as champions for Lassa fever communication allowed release of funds for sensitization in LGAs, increased awareness and acceptance - Collaboration between States, NGOs, and partners to reach larger community on Lassa Fever awareness and sensitization led to increase awareness, positive behavioural change - Formation of community observers and monitors to ensure adherence to positive food handling and environmental sanitation led to community ownership and sustainability |

Prioritised Activities 2018 AAR: Risk Communication and Social Mobilization:

1. Train social mobilization officers across LGAs in states

2. Production of IEC material

3. Media engagement

### Supplementary Material 2. Results South Sudan IAR 2020 to 2021

**Surveillance, case investigation and contact tracing**

| **Challenges 2020** | **Identified Recommendations 2020** | **Best Practice Actions Observed 2021** |
| --- | --- | --- |
| Inconsistent attendance by decision makers affected communication between the implementers decision  makers leading to expanded workdays and thus requiring additional time to be spent in follow up meetings. | For immediate implementation:   - Build DHIS2 and complete the COVID-19 component for full data capture - Enrolment of states hospitals into the SS sites for COVID - Activate community surveillance via Boma Health Initiative - Cross-border surveillance planning - Train the frontline health workers and community health workers on early detection, reporting, quarantine of suspect COVID-19 cases - Deploy IDSR and EWARS resources including surveillance focal points at health facility; county; state; and national level (HF SFP; CSOs, SSOs, EPR department), RRTs, and EWARS electronic platform for the detection, reporting, investigation, and responding to COVID-19 suspect and confirmed cases - Continue implementation of community-based contact tracing as a model for engaging communities and improving compliance to contact listing and follow up - Maintain and reinforce surveillance for IDSR priority diseases; vaccine preventable diseases like malaria; polio, and measles and deliver routine immunization and other cost effective public health interventions using existing health system resources - Rollout training on the third edition IDSR Technical Guidelines to the counties to strengthen capacities for surveillance and response to IDSR priority diseases, and other public health emergencies including COVID-19.   For mid to long-term implementation to improve the response to the ongoing COVID-19 outbreak:   - Establish good communication system for COVID data flow - Verifying and cleaning data for COVID - Leverage existing electronic system nationwide for collecting data | - Availability of surveillance system –Early Warning, Alert and Response System (EWARS) for Acute Respiratory Tract Infection (ARI) alert reporting and verification. - Regular daily and weekly briefings are undertaken by the COVID-19 response team. - Contact tracing formation and activation; teams are available to conduct contact listing and follow-up. - Coordination between National RRT/National Public Health Laboratory (NPHL) with State RRT lead to improved reporting and information sharing between the NRRT, NPHL and SRRT and therefore improved engagement with the states. |
| Communications across pillars especially the laboratory with late or missing test results, challenges in using unique identifiers and the limited testing capacities in the country at the peak of the outbreak. | - Establish good communication system for COVID data flow - Leverage existing electronic system nationwide for collecting data | - Availability of COVID-19 guidelines, SOPs, and protocols which allowed the contact tracing team and RRT to timely response investigate and follow up confirmed cases and their contacts. |

1. AAR Lassa Fever Nigeria (21-22 August 2017), p 13-14 [↑](#footnote-ref-1)
2. AAR Lassa Fever Nigeria (5-7 June 2018), p 14-15 [↑](#footnote-ref-2)
3. AAR Lassa Fever Nigeria (21-22 August 2017), p 13-14 [↑](#footnote-ref-3)
4. AAR Lassa Fever Nigeria (21-22 August 2017), p 13-14 [↑](#footnote-ref-4)
5. AAR Lassa Fever Nigeria (5-7 June 2018), p 15-16 [↑](#footnote-ref-5)
6. AAR Lassa Fever Nigeria (21-22 August 2017), p 13-14 [↑](#footnote-ref-6)
7. AAR Lassa Fever Nigeria (21-22 August 2017), p 13-14 [↑](#footnote-ref-7)
8. AAR Lassa Fever Nigeria (5-7 June 2018), p 15-16 [↑](#footnote-ref-8)
9. AAR Lassa Fever Nigeria (21-22 August 2017), p 13-14 [↑](#footnote-ref-9)
10. AAR Lassa Fever Nigeria (21-22 August 2017), p 13-14 [↑](#footnote-ref-10)
11. AAR Lassa Fever Nigeria (5-7 June 2018), p 15-16 [↑](#footnote-ref-11)
12. AAR Lassa Fever Nigeria (21-22 August 2017), p 13-14 [↑](#footnote-ref-12)
13. AAR Lassa Fever Nigeria (21-22 August 2017), p 13-14 [↑](#footnote-ref-13)
14. AAR Lassa Fever Nigeria (5-7 June 2018), p 15-16 [↑](#footnote-ref-14)
